# Comparative Methylome Analysis Reveals Perturbation of Host Epigenome in Chestnut Blight Fungus by a Hypovirus

**DOI:** 10.3389/fmicb.2018.01026

**Published:** 2018-05-23

**Authors:** Ru Li, Sisi Zhou, Yongbing Li, Xiaorui Shen, Zhiqiang Wang, Baoshan Chen

**Affiliations:** ^1^State Key Laboratory for Conservation and Utilization of Subtropical Agro-bioresources, Guangxi University, Nanning, China; ^2^Department of Biotechnology, College of Life Science and Technology, Guangxi University, Nanning, China

**Keywords:** *Cryphonectria parasitica*, hypovirus, methylome, RNA-Seq, virulence

## Abstract

In eukaryotic genomes, DNA methylation is an important type of epigenetic modification that plays crucial roles in many biological processes. To investigate the impact of a hypovirus infection on the methylome of *Cryphonectria parasitica*, the chestnut blight fungus, whole-genome bisulfite sequencing (WGBS) was employed to generate single-base resolution methylomes of the fungus with/without hypovirus infection. The results showed that hypovirus infection alters methylation in all three contexts (CG, CHG, and CHH), especially in gene promoters. A total of 600 differentially methylated regions (DMRs) were identified, of which 144 could be annotated to functional genes. RNA-seq analysis revealed that DNA methylation in promoter is negatively correlated with gene expression. Among DMRs, four genes were shown to be involved in conidiation, orange pigment production, and virulence. Taken together, our DNA methylomes analysis provide valuable insights into the understanding of the relationship between DNA methylation and hypovirus infection, as well as phenotypic traits in *C. parasitica*.

## Introduction

In eukaryotes, DNA methylation is an important epigenetic modification mechanism that is involved in many cellular processes such as genomic imprinting, gene expression regulation, cellular differentiation, genome integrity, and disease development (Bird, 2002; Suzuki and Bird, 2008; Conerly and Grady, 2010; Kulis and Esteller, 2010). Recently, advances in whole genome bisulfite sequencing (WGBS) have generated single-base resolution methylomes of more than 20 eukaryotic organisms, including invertebrates, vertebrates, and plants. In these studies, many elaborate methylation patterns and functional roles of DNA methylation have been revealed (Lister et al., 2008, 2009; Zemach et al., 2010; Zhong et al., 2013; Wang X. et al., 2014).

Characteristics of DNA methylation have also been reported on fungi and it was found that the degree, distribution, and function of DNA methylation varied greatly among fungal species (Zemach et al., 2010). For example, the *Neurospora* genome is methylated by 1.5%, whereas DNA methylation in *Aspergillus flavus* is negligible (Foss et al., 1993; Liu et al., 2012). In *Neurospora*, DNA methylation is mainly regarded as a genome defense mechanism to silence transposable elements and DNA repeats (Martienssen and Colot, 2001; Selker et al., 2003). In *Candida albicans*, DNA methylation takes place predominantly in structural genes and regulates transcriptional activity, with repeat regions largely devoid of methylation (Mishra et al., 2011). In *Magnaporthe oryzae*, DNA methylation serves as a dynamic epigenetic modification functioning in genome defense and fungal development (Jeon et al., 2015) and DNA methylation was shown to be a dynamic process during sexual development in *Cordyceps militaris* (Wang et al., 2015). However, relationship between DNA methylation and virulence regulation has not been reported in fungi.

*Cryphonectria parasitica* is the causal agent of chestnut blight disease and hypovirulence caused by hypovirus infection has been used to probe fungal pathogenicity/virulence regulation (Dawe and Nuss, 2001). Wild-type *C. parasitica* strain EP155 can incite big cankers on chestnut stems and is with orange pigmentation and normal filamentous hyphae on PDA plate, produces abundant asexual conidial spores and is able to produce sexual spores through mating with opposite sexual strain. Hypovirus infection profoundly reduces virulence and causes pleiotropic effects in multiple traits, including reduced colony growth rate and pigmentation, diminished asexual spore production, and suppression of female sterility to its host fungus (Nuss, 2005). Transcriptional analysis based on EST (Dawe et al., 2003; Shang et al., 2008) and cDNA microarray (Allen et al., 2003) revealed significant impacts of hypovirus infection on the host gene expression. Recently, comparative proteomic analysis also showed selective regulation of host protein expression by hypovirus (Wang et al., 2013) and two DNA methyltransferases were found to be up-regulated significantly in the hypovirus-infected strain EP155/CHV1-EP713 (Wang J. et al., 2014). Deletion of *sahh*, a hypovirus-regulated gene encoding S-adenosyl-homocysteine hydrolase, resulted in the elevated accumulation of intracellular SAM (a methyl donor), and a significant reduction in virulence (Liao et al., 2012). These findings imply that hypovirus may perturb the methylation pattern of the fungus to modulate fungal virulence and other important traits.

In this study, the impact of hypoviral infection on DNA methylation in *C. parasitica* was investigated by generating and comparing two DNA methylomes at a single-base resolution using BS-seq and the link between DNA methylation and gene expression was explored via transcriptional profiling. Furthermore, the roles of several differentially methylated regions (DMRs) associated genes were functionally examined. Our results provide new insights into the relationship between hypovirulence and DNA methylation.

## Materials and methods

### Fungal strains and culturing conditions

The *C. parasitica* strains used in this study were the wild-type strain EP155 (ATCC 38755), its isogenic strain EP155/CHV1-EP713 (synthetic hypovirus CHV1-EP713 infected EP155) (Chen et al., 1994), and a highly efficient homologous recombination strain KU80 (Δ*ku80* of EP155,) (Lan et al., 2008). The fungal strains were maintained on potato dextrose agar (PDA) at 24–26°C with a 12 h light (1,500 1x) and 12 h dark cycle (Hillman et al., 1990). Cultures used for DNA and RNA extraction were grown on PDA medium for 7 days. Protoplasts of *C. parasitica* stain KU80 were prepared and transformed as described previously, with hygromycin (40 μg/mL) complemented in the medium for transformant selection (Churchill et al., 1990; Chen et al., 2011). For morphological characterization, strains were inoculated onto PDA plates and maintained for up to 14 days to allow sporulation.

### BS-seq library construction and high-throughput sequencing

Total genomic DNA were extracted using the method previously described (Churchill et al., 1990). For library construction, DNA from two independently cultured mycelium samples of a strain was mixed equally and a total amount of 5.2 microgram genomic DNA spiked with 26 ng lambda DNA were fragmented by sonication to 200–300 bp with Covaris S220 (Covaris, Woburn MA, USA), followed by end repair, adenylation, and ligation of cytosine-methylated sequencing adapters as manufacturer's instructions. The bisulfite conversion was performed using an EZ DNA Methylation-Gold^TM^ kit (Zymo Research). Following PCR amplification, amplicons were quantified and insert sizes were confirmed. The library preparations were sequenced using an Illumina HiSeq 2000/2500 platform, with raw sequencing data processed within the standard Illumina pipeline according to previously reported methods (Jeon et al., 2015).

### Read processing and alignment

Raw reads from high-throughput sequencing were first preprocessed and quality control was assessed using in-house Perl scripts. Reads with adaptor sequences, low-quality, or those containing more than 10% Ns (unknown bases) were removed to obtain high-quality clean reads. The obtained bisulfite-treated reads were then aligned to the *C. parasitica* genome (http://genome.jgi-psf.org/Crypa2/Crypa2.home.html) using Bismark software v 0.12.5 (Krueger and Andrews, 2011). First, the reference genome was transformed into a bisulfite-converted version (C-to-T and G-to-A converted), with sequence reads also transformed. The similarly converted genomic versions were then aligned in a directional manner. Two alignment processes (original top and bottom strands) were employed and the obtained unique best alignment was then compared to the normal genomic sequence, with the methylation state of all cytosine positions in the read inferred. The uniquely mapped reads were also used to compute the sequencing depth and coverage. The sodium bisulfite non-conversion rate was calculated as the percentage of cytosines sequenced at cytosine reference positions in the lambda genome as previously reported (Hao et al., 2016).

### Identification of differentially methylated regions

The swDMR software (https://sourceforge.net/projects/swdmr/) which utilizes a sliding-window approach was used to identify differentially methylated regions (DMRs) as previously described (Hao et al., 2016). Only cytosines with a depth of at least four in all libraries were used. A window size of 1000 bp with a step length of 100 bp was used in this analysis. For each window, the methylation level at each cytosine was analyzed for each of the two samples. A Fisher test was then performed for each window. The resulting *P*-values were corrected for multiple tests with a false discovery rate (FDR). Regions with a corrected *P*-value < 0.05 and changes of methylation level of at least two-fold were identified as DMRs.

### RNA-seq analysis

Total RNAs extracted from two independently cultured mycelium samples of a strain was mixed in equal amount for cDNA library construction using NEBNext® Ultra™ RNA Library Prep Kit (NEB, USA). The libraries were sequenced with a Illumina HiSeq platform following the manufacturer's instructions. Prior to mapping reads to the reference database, raw reads were processed to remove adaptor sequence, low quality reads and reads containing ploy-N. All clean reads were aligned to the *C. parasitica* reference genome using TopHat v2.0.12. HTSeq v0.6.1 was applied to count the reads numbers mapped to each gene, and the FPKM (expected number of Fragments Per Kilobase of transcript sequence per Millions mapped reads) values were used for quantification of gene expression level (Mortazavi et al., 2008). Prior to differential gene expression identification, the read counts for each library were adjusted using edgeR program package through one scaling normalized factor. We used the DEGseq R package (1.20.0) to analyze the differential expression genes (DEGs) between EP155 and EP155/CHV1-EP713. The *P*-values were adjusted by the Benjamini and Hochberg's method (Anders and Huber, 2010). We used a corrected *P*-value of ≤0.005 and log_2_ (fold change) ≥1 as the criteria to judge the significance of gene expression difference.

### Confirmation of BS-seq and RNA-seq results

DNA sample of 1 μg was bisulfite-converted as previously reported (Espada et al., 2014) and primers were designed to amplify target DMRs (Table S1). Purified amplicons were cloned into pMD-18T vectors (Takara) and a minimum of ten independent clones were analyzed for each target region. The relative accumulation of gene transcripts was evaluated using quantitative real-time RT-PCR (Lin et al., 2007), with primers specific for the targeting genes (Table S1) and 18S rRNA was used as a normalization reference. For each of the examined genes, 3 independent replicates were performed.

### Construction of gene deletion mutants

Targeted gene deletion was performed by homologous recombination, using a hygromycin B resistance (*hph*) cassette to replace the targeted gene in *C. parasitica* as described previously (Lan et al., 2008). In the case of *Tpk* gene, the 990-bp 5′ and 890-bp 3′ flanking regions were amplified with the Tpk-left and Tpk-right primers using total DNA from EP155 as template, respectively. Primer left-reverse contains 26 nucleotides identical to the 5′-end of the *hph* cassette, and primer right-forward contains 26 nucleotides identical to the 3′-end of the *hph* cassette. The *hph* cassette was amplified using plasmid pCPXHY2 as template with primers Hyg-forward/Hyg-reverse. The 990-bp 5′ flanks region, the 2,146-bp *hph* cassette and the 890-bp 3′ flanks region were joined to form a 4.0-kb cassette by fusion PCR. After verification by agarose gel electrophoresis and gel extraction, the PCR product was resuspended in TE buffer to a final concentration of 1 μg/μL and used to transform KU80 protoplasts as described previously. Putative *Tpk* disruptants were identified by PCR with primers Tpk-all, and purified to nuclear homogeneity by single-spore isolation. Confirmed transformants were designated as Δ*Tpk* strains. The detailed methods used to generate mutants Δ*Abh*, Δ*Met*, Δ*Stk* generation are similar to that described above. Gene cloning and PCR analysis (detailed primer sequences are listed in Table S1) were performed according to Sambrook and Russell (2001).

### Pathogenicity assay

Fungal pathogenicity was analyzed using dormant stems of Chinese chestnut (*Castanea mollissima*) according to Shi et al. (2014). For each fungal strain, 3 replicates were performed. After inoculation, the stems were incubated in a plastic bag at 25°C to allow lesion development. Canker sizes were measured and analyzed 25 days after inoculation.

## Results

### Hypovirus infection changes the DNA methylation profile of the fungal genome

To generate a genome-wide DNA methylation map of *C. parasitica*, DNA was extracted from the mycelia and high throughput whole-genome bisulfite sequencing (WGBS) was performed. With unmethylated lambda DNA as a reference to calculate the conversion rate (99.97% for both samples), 13,502,819 and 13,948,110 raw reads were obtained for the wild type strain EP155 and its isogenic virus-infected strain EP155/CHV1-EP713, respectively. After trimming off low-quality reads and retaining unique mapped reads, 7,183,382 and 8,586,367 reads were used for further analysis. The read depths ranged from 16.21× to 19.38× per base for each DNA strand, with more than 96% of cytosine covered by at least five sequencing reads (Table S2).

**Figure 1:**
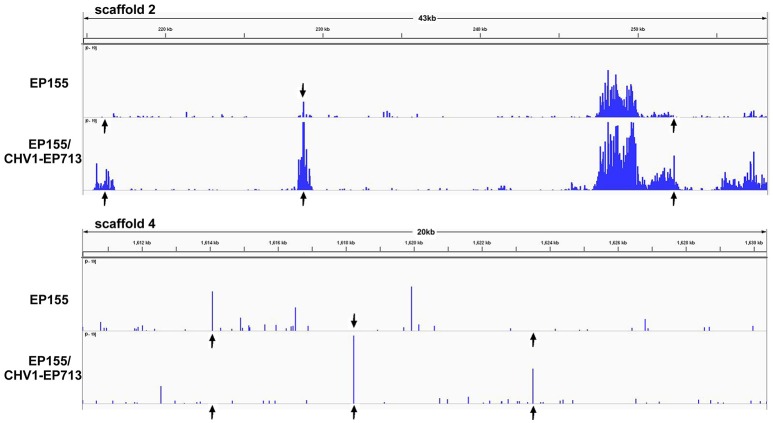
Distribution of DNA methylation in the genome of *C. parasitica*. The identified methylcytosines densities on scaffold 2 and scaffold 4 are shown. Arrows indicate representative regions showing dynamic changes in DNA methylation between EP155 and EP155/CHV1-EP713.

In virus-free strain EP155, 1.02% of methylation (0.04% at CG, 1.20% at CHG, and 1.30% at CHH sites) at the genome-wide scale was detected based on WGBS data, compared with 1.14% (0.04% at CG, 1.38% at CHG, 1.44% at CHH sites) for virus-infected EP155/CHV1-EP713 (Table 1). Although no significant differences were observed in overall methylcytosine (mC) percentages, many variations were found in the distribution of mC between the two genomes (Figure 1), indicating that DNA methylation of *C. parasitica* were changed in response to hypovirus infection.

**Table 1:** DNA methylation levels in EP155 and EP155/CHV1-EP713.

**Sample**	**C_rate (%)**	**CG_rate (%)**	CHG_rate^*^ (%)	CHH_rate^*^ (%)
EP155	1.02	0.04	1.20	1.30
EP155/CHV1-EP713	1.14	0.04	1.38	1.44

* *H = A, T, or C*.

**Figure 2:**
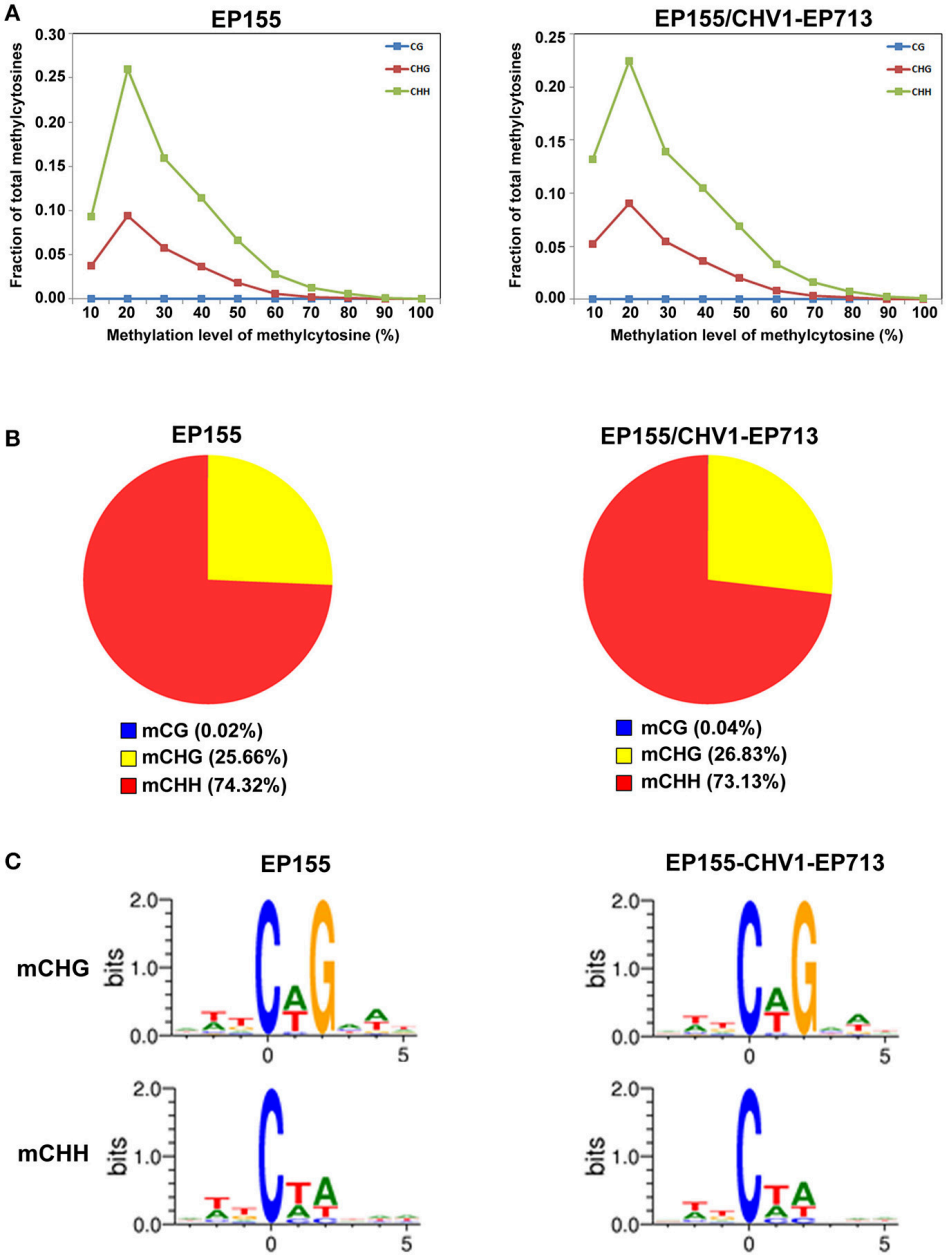
The global pattern of EP155 and EP155/CHV1-EP713 DNA methylomes. **(A)** Distribution of mCs in each sequence context (H = A, T, or C) in EP155 (Left) and EP155/CHV1-EP713 (Right). Methylation levels on the *x*-axis are defined as the percentage of reads showing mC cytosine at a reference cytosine site. The y-axis shows the fraction of total mCs calculated within bins of 10%. **(B)** The percentage and absolute number of mCs identified in EP155 (left) and EP155/CHV1-EP713 (right) in three sequence contexts. **(C)** Logo plots of the sequences proximal to sites of non-CG DNA methylation in each sequence context in EP155 (left) and EP155/CHV1-EP713 (right).

When examining mC distribution, higher methylation levels were seen at mCHH and mCHG sites relative to mCG (Figure 2A). Moreover, methylated sites were found to be concentrated in the non-CG sites, especially mCHH (Figure 2B), with basic group HH or mCHH tending to comprise two-fold more A or T (Figure 2C). Additionally, the mC distribution was noted to be in high mC percentage in scaffolds 12–21 and 24–25 across *C. parasitica* chromosomes (Figure S1).

**Figure 3:**
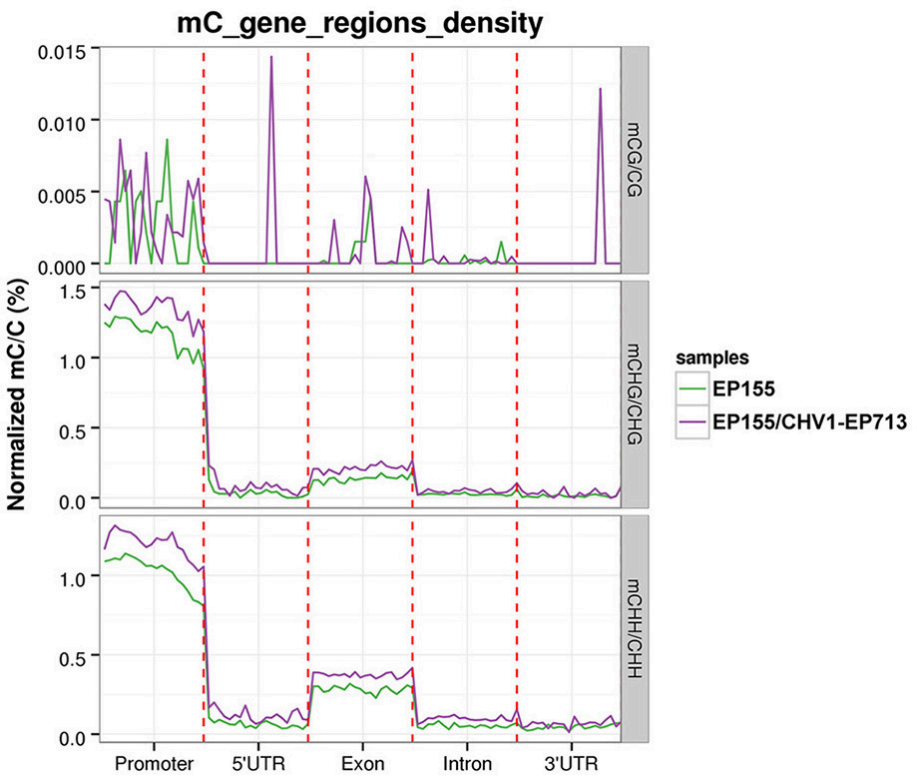
Density of mCs in different genomic features in EP155 and EP155/CHV1-EP713. The *y-* axis indicates the mCs densities for each element, with the genomic regions listed on the *x*-axis, which include the promoter (2,000 bp upstream of the transcriptional start sites), 5′UTR, exon, intron, and 3′UTR.

By calculating and comparing DNA methylation levels at regions of promoters, untranslated regions (UTRs), exons and introns, it was revealed that the methylation levels in promoters were significantly higher than in gene-body regions (UTRs, exons and introns); in the gene body, the methylation levels in exons were higher than those in introns, 5′- and 3′-UTRs. Furthermore, the methylation levels of the three C contexts in EP155/CHV1-EP713 were always higher than those in EP155, especially in promoters (Figure 3).

To investigate the relation of DNA methylation pattern and hypovirus infection, differentially methylated regions (DMRs) of EP155 and EP155/CHV1-EP713 were searched and a total of 600 DMRs were identified (Table S3). Among the 144 DMRs with gene annotation, 89 (61.8%) were located in promoters and 17 (11.8%) in gene bodies (Table 2 and Table S4). Moreover, 79 hypermethylated genes were identified in EP155/CHV1-EP713 and 65 in EP155, indicating that hypovirus infection changes DNA methylation level of its host. To obtain gene expression profiles for the two strains, 27,560,950 and 27,003,506 raw reads were generated for EP155 and EP155/CHV1-EP713, respectively, by RNA-seq and 24,060,546 and 11,727,947 reads, respectively, were uniquely mapped to the *C. parasitica* reference genome (Table S5). A total of 2,717 (1207 genes up- and 1510 down-regulated) differentially expressed genes were found in EP155/CHV1-EP713, as compared with EP155 (Table S6). Gene Ontology enrichment analysis unveiled that the differentially expressed genes were markedly enriched in oxidoreductase activity and catalytic activity domains and KEGG pathway analysis demonstrated that metabolic pathways were enriched significantly (Figure S2).

**Table 2:** Distribution of DMRs in EP155 and EP155/CHV1-EP713.

	**Total**	**Promoter**	**Gene body**	**Promoter and Gene body**
Total	144	89	17	38
Hyper^a^	79	42	5	32
Hypo^b^	65	47	12	6

a *Hypermethylation in EP155/CHV1-EP713*.

b *Hypomethylation in EP155/CHV1-EP713*.

To validate the accuracy of the WGBS, bisulfite-PCR/sequencing were performed for three randomly selected DMRs in both EP155 and EP155/CHV1-EP713. As shown in Figure S3, results of bisulfite-PCR matched well with those of WGBS.

### Relationship between DNA methylation and gene expression

**Figure 4:**
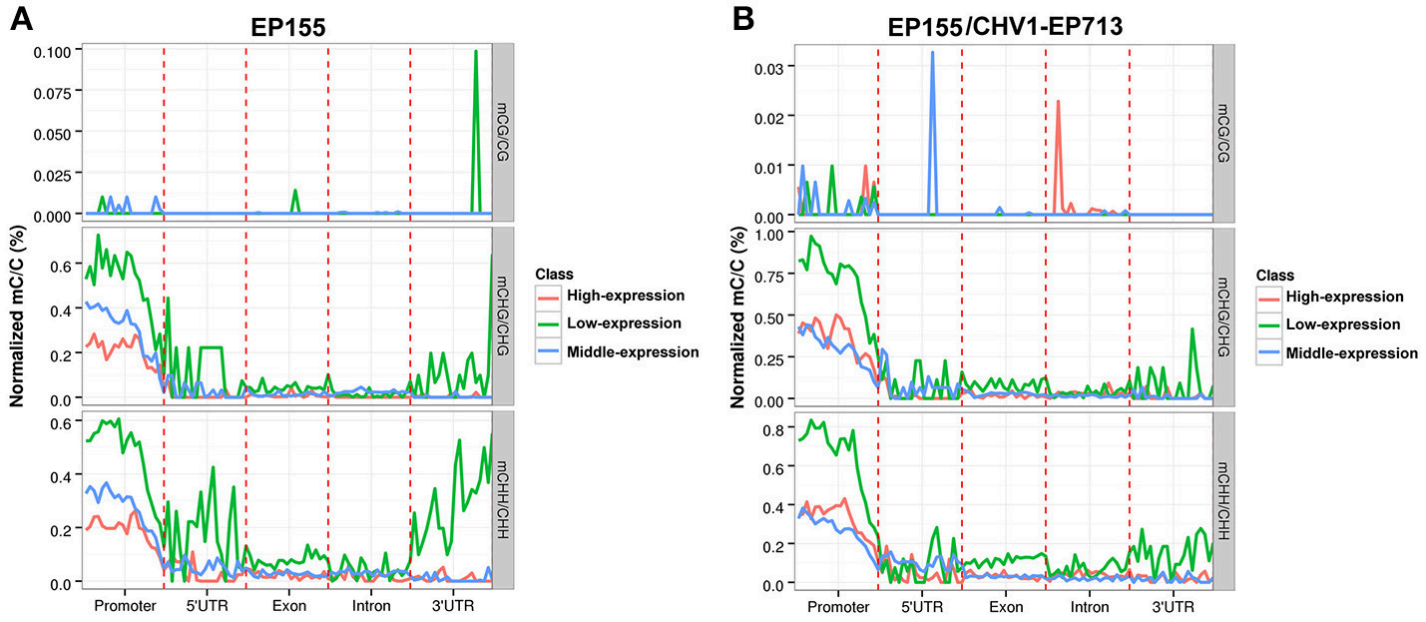
Relationship between DNA methylation and gene expression in *C. parasitica*. **(A)** The methylation level trend (*y-* axis) of three different gene clusters (Genes were classified into three categories based on expression levels: high, medium, and low expression, from the bottom one-third to the top one-third) in genomic regions (*x*-axis) in EP155. **(B)** The methylation level trend (*y-* axis) of three different gene clusters in genomic regions (*x*-axis) in EP155/CHV1-EP713.

We evaluated the impacts of *C. parasitica* DNA methylation on gene expression via RNA-seq. RNA-seq results indicated that low-expression genes (bottom one-third) had significantly higher methylation levels in their promoters than high-expression genes (top one-third), suggesting that DNA methylation in promoter has negative correlation with gene expression. Meanwhile, methylation level in gene body seemed not to have a clear correlation with gene expression (Figure 4). The accuracy of the RNA-Seq results were validated by qRT-PCR in which six randomly selected DMR-associated genes revealed by RNA-Seq were examined (Figure S4).

### Functional analysis of DMR-associated genes

**Figure 5:**
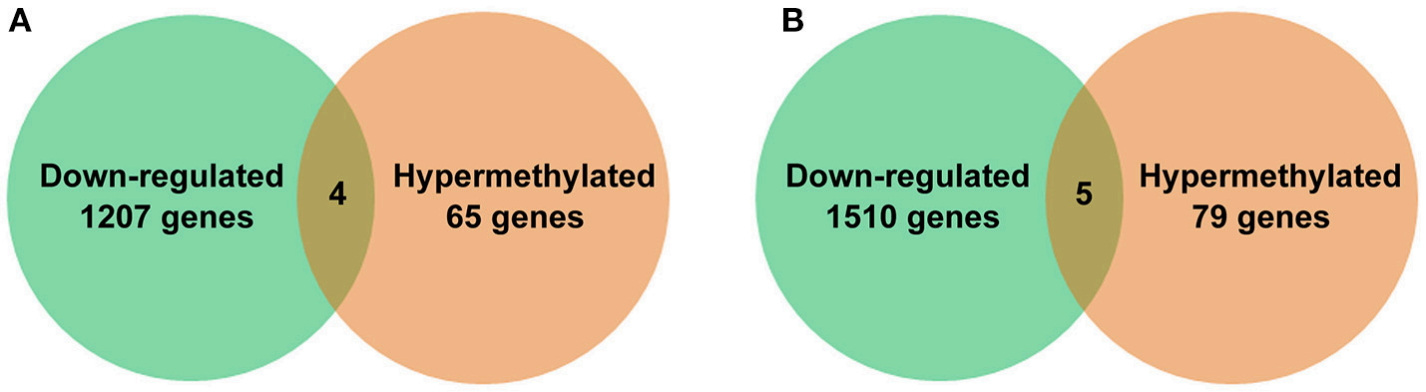
Down-regulated genes and hypermethylated DMR-associated genes in *C. parasitica*. The number of down-regulated genes and hypermethylated DMR-associated genes in **(A)** EP155 and **(B)** EP155/CHV1-EP713.

**Figure 6:**
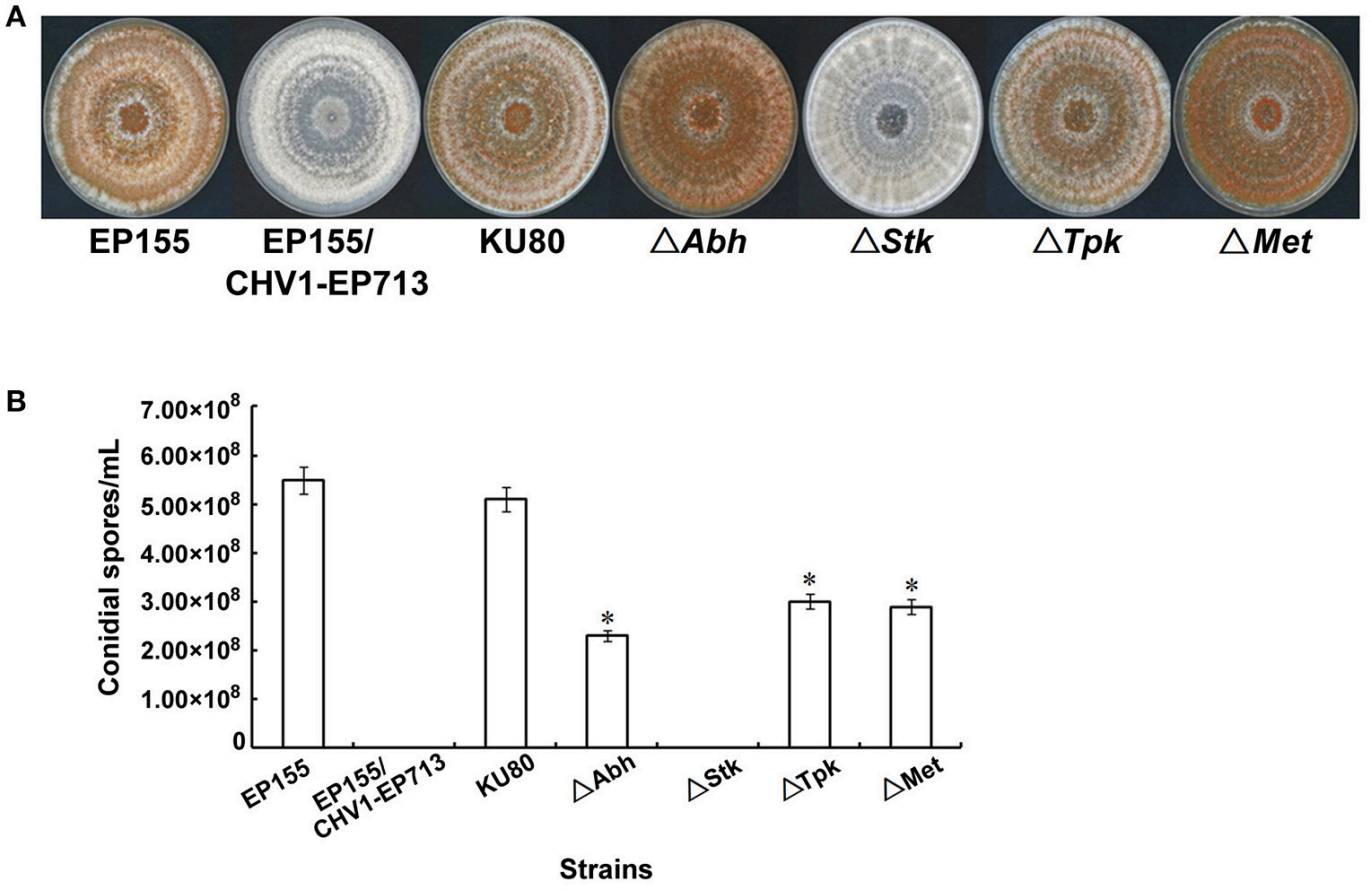
Phenotypes of four DMR-associated gene deletion mutants. **(A)** Colonies on PDA plates at day 14 post-inoculation. All mutants were indistinguishable in the colony growth rate from EP155 and KU80. While mutants Δ*Tpk*, Δ*Abh* and Δ*Met* were all with orange pigment, Δ*Stk* strains was almost white in colony. **(B)** Sporulation characteritics of the indicated strains. Asterisk indicates statistical significance relative to EP155 (*P* < 0.01; *t*-test). All mutants were impaired in sporulation, and Δ*Stk* failed to produce any spores although the culturing time was extended to 20 days.

DNA methylation is generally considered as a silencing epigenetic modification and thus genes down-regulated at transcriptional level after methylation would likely be a result of hypermethylation. By comparison of methylation patterns and transcriptomes, four down-regulated genes were identified among 65 hypermethylated DMRs in EP155, and five down-regulated genes were identified among 79 hypermethylated DMRs in EP155/CHV1-EP713 (Figure 5). Among these nine genes, five were annotated to have clear function: tyrosine protein kinase (Tpk), alpha/beta hydrolase (Abh), S-adenosyl-L-methionine-dependent methyltransferase (Met), serine/threonine protein kinase (Stk), and chromodomain-helicase DNA-binding protein (Chd) (Table 3). To further investigate the relationship between DNA methylation and gene expression, we tried to disrupt these genes one by one by gene replacement. A total of 8, 6, 10, and 5 of verified knockout mutants for genes Tpk, Abh, Met, and Stk were obtained and representative null mutants were subjected to phenotypic characterization. When cultured on PDA plates at 24–26°C for 14 days, all mutants were indistinguishable in the colony growth rate from the wild-type strain EP155 and original strain KU80. Furthermore, no obvious difference in the hypha morphology was observed between the mutants, EP155, and KU80 (Figure S5). While mutants Δ*Tpk*, Δ*Abh* and Δ*Met* were all with orange pigment, Δ*Stk* strains was almost white in colony. All mutants were impaired in sporulation, and Δ*Stk* failed to produce any spores (Figure 6).

**Table 3:** Down-regulated and hypermethylated DMR-associated genes in EP155 and EP155/CHV1-EP713.

**Gene_id**	**DMR location**	**Annotation**	**Accession number**
**EP155**			
estExt_Genewise1Plus.C_20119	Promoter	Tyrosine protein kinase	286354
Crypa1.e_gw1.18.38.1	Promoter	Alpha/beta hydrolase	53336
Crypa1.fgenesh1_pg.C_scaffold_14000121	Promoter	Hypothetical protein	75832
fgenesh1_kg.7_#_296_#_CEST_15_H_03	Promoter	Hypothetical protein	323714
**EP155/CHV1-EP713**			
fgenesh1_pg.5_#_525	Promoter	S-adenosyl-L-methionine-dependent methyltransferase	330939
e_gw1.2.2145.1	Promoter	Serine/threonine protein kinase	251174
Crypa1.fgenesh1_pg.C_scaffold_2000113	Promoter	Chromodomain-helicase DNA-binding protein	67838
Crypa1.fgenesh1_pg.C_scaffold_15000088	Promoter	Hypothetical protein	75956
Crypa1.estExt_fgenesh1_pg.C_120009	Promoter	Hypothetical protein	109204

**Figure 7:**
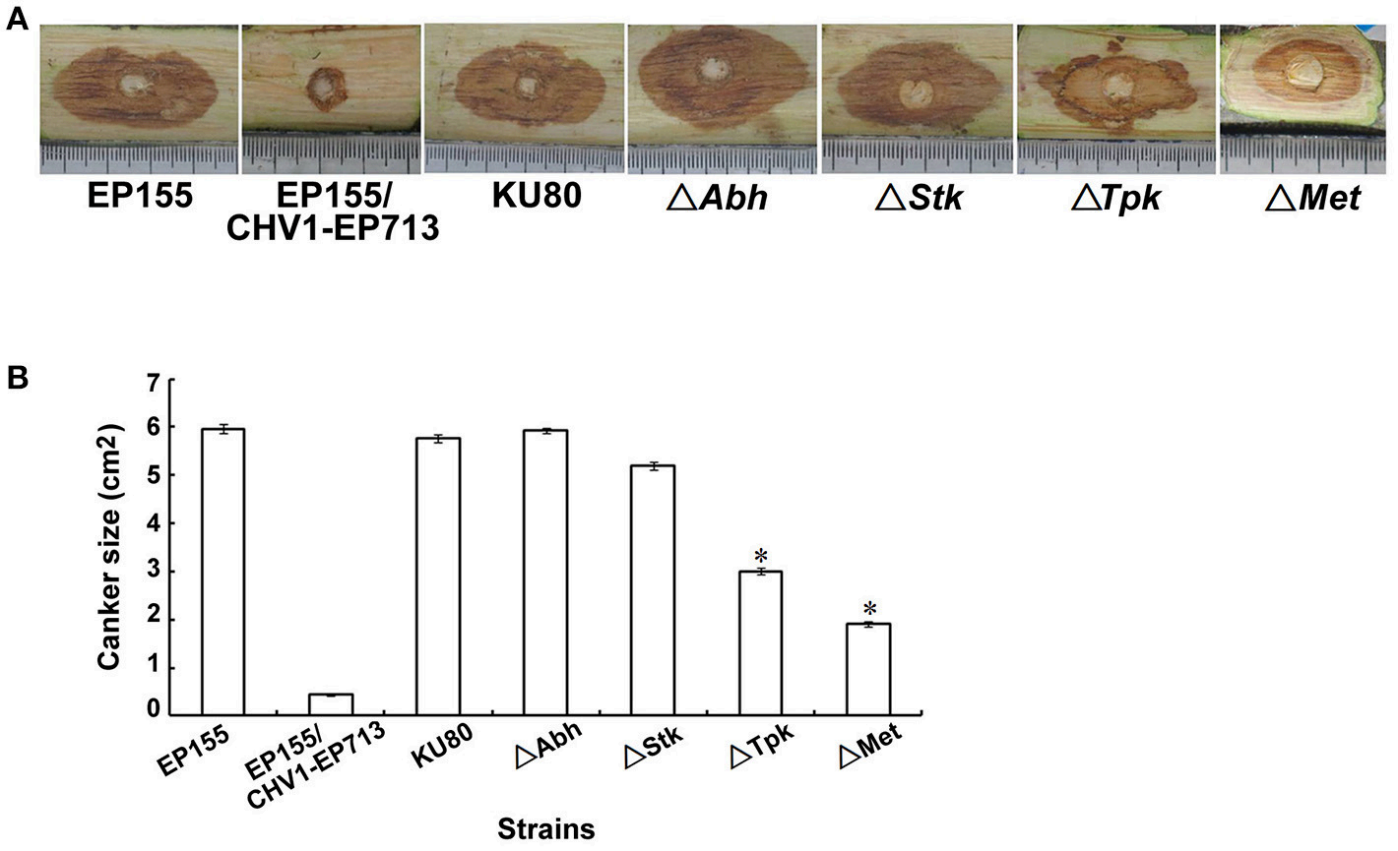
Pathogenicity assay of the indicated strains on Chinese chestnut stems. **(A)** Dormant Chinese chestnut (*Castanea mollissima*) stems were inoculated and maintained at 25°C. The cankers were measured and photographed at day 25 post-inoculation. **(B)** Canker size measurements of the tested strains. The assays were performed in triplicate for each strain, with an asterisk indicating a statistical significance relative to EP155 (P < 0.01; *t*-test).

The pathogenicity of four genes deletion mutants was further analyzed on dormant chestnut stems. As shown in Figure 7, the wild-type strain EP155 and KU80 were highly virulent, whereas the hypovirus-infected strain EP155/CHV1-EP713 produced much smaller cankers. Compared with EP155 and KU80, Δ*Abh* and Δ*Stk* showed no significant change in virulence, while Δ*Tpk* and Δ*Met* were attenuated in virulence with canker size about 1/3-1/2 of the wild-type strain. These results indicate that *Tpk* and *Met* genes contribute to the virulence of *C. parasitica*, but not *Abh* and *Stk*.

## Discussion

Toward elucidation of mechanism of hypovirulence by a hypovirus, three main cellular processes, signal transduction (Nuss, 1996), vesicular secretory pathways (Kazmierczak et al., 2012; Wang et al., 2013, 2016) and primary metabolism (Allen et al., 2003; Dawe et al., 2009) have been shown to be perturbed by hypovirus infection. Consistent with these studies, RNA profiling in this study showed significant effects of hypovirus infection on the expression of host genes which function in catalytic activity and metabolic pathways (Figure S2). It has been reported that the metabolic state of the cell can affect DNA methylation through a variety of mechanisms: (i) the expression or activity of the enzymes involved in cytosine methylation can be affected by metabolic signaling; (ii) metabolites can modulate DNA methylation by influencing the function/localization of proteins that recruit or regulate DNA-modifying enzymes; (iii) cellular metabolites serve as the substrates and cofactors for DNA-modifying enzymes, and alterations of these metabolite levels can in turn result in global changes of DNA methylation patterns (Sharma and Rando, 2017). Therefore, we propose that hypovirus may perturb the DNA methylation pattern of its host through regulation of the metabolic state of *C. parasitica*.

While the DNA methylation level is higher in plants and mammals, fungi generally have lower level of methylation, ranging from negligible to just barely detectable (Lister et al., 2008, 2009; Liu et al., 2012; Li et al., 2017). DNA methylation in *C. parasitica* was determined to be at level of 1.02% (methylated cytosines) in the wild-type strain EP155 and 1.14% in the hypovirus-infected strain EP155/CHV1-EP713 (Table 1) in this study, consistent with the methylation levels reported for other fungi.

Earlier studies suggested that infection by Epstein-Barr virus induces widespread demethylation of the host genome (Hansen et al., 2014) and human herpesvirus 6B induces hypomethylation on chromosome 17p13.3 (Engdahl et al., 2017), showing a complex epigenetic modification effects upon viral infection. Although global methylation levels were not significantly different between EP155 and EP155/CHV1-EP713, clear alterations in methylation pattern were identified in the genome of *C. parasitica* after viral infection (Figure 1). Furthermore, DNA methylation levels in the promoters and gene-body regions were generally higher in EP155/CHV1-EP713 than in EP155, suggesting that hypovirus infection may affect a basal enzymatic activity for methylation.

Among the 600 DMRs identified in *C. parasitica*, more than three quarters were located in the intergenic regions, consistent with that observed in *C. militaris* (Wang et al., 2015) and soybean (Song et al., 2013), but in sharp contrast with *Arabidopsis* and tomato, in which the DMR enrichment was found to localize in genic regions (Becker et al., 2011; Zhong et al., 2013).

DNA methylation is generally considered as a silencing epigenetic modification (Robertson, 2005). However, recent studies suggest that the relationship between DNA methylation and gene transcription is far more complicated than previously reported (Jones, 2012). In this study, RNA-Seq revealed that methylation in promoter negatively correlated with transcript abundance of a gene in general, while methylation in gene body did not have a clear correlation (Figures 4, 5), similar to the phenomena previously observed in rice, *Arabidopsis* and *C. militaris* (Vaughn et al., 2007; Li et al., 2012; Wang et al., 2015). It is speculated that gene expression was regulated by DNA methylation, chromatin modification and genetic changes in *cis*- or *trans*-regulators.(Li et al., 2012).

By loss-of-function analysis, we demonstrated that some of the DMR-associated genes were functionally important to the fungal sporulation, orange pigmentation, and/or virulence (Figures 6, 7). These genes are predicted to encode protein kinases, alpha/beta hydrolase, and methyltransferase and they were all down-regulated at transcriptional level after methylation (Table 3). Relevant to our findings, previous studies have suggested that signal transduction components may be perturbed by hypovirus infection to induce hypovirulence (Turina and Rostagno, 2007). Several of these genes constitute the G-protein signaling cascades while others function in mitogen-activated protein kinase (MAPK) pathways (Rostagno et al., 2010). MAPKs of Ser/Thr kinases have been shown to be required for fungal growth, development, and pathogenicity (Park et al., 2012). In the present study, we demonstrated that hypovirus infection resulted in hypermethylation and down-regulation of a Ser/Thr protein kinase gene (*Stk*), and found that the methylated gene *Stk* was essential for sporulation and orange pigmentation. This is in consistent with the observation that disruption of *cpmk1* and *cpmk2*, which encode MAPKs of *C. parasitica*, result in reduced pigmentation and conidiation (Park et al., 2004; Choi et al., 2005). Our results indicate that hypovirus may change fungal phenotypic traits by selectively regulating DNA methylation and transcription of genes. Further analysis of DNA methylation of more protein kinase genes and testing the interaction of methylation and gene expression will help to reveal the precise mechanism by which hypovirus regulates host signal transduction pathways.

Tyrosine protein kinases (TPKs) play a crucial role in signal transduction and also in cell growth, differentiation, death and a series of cellular processes in animals. Mutations of TPKs can cause various human diseases, such as cancer and immune diseases (Jiao et al., 2018). It is reported that fungi lack orthologs of animal TPK, but they have a specific lineage of protein kinases closely related to TPKs. (Zhao et al., 2014). To date, their potential roles in fungi are still largely unknown. Our results showed that *Tpk* of *C. parasitica* functions in the regulation of virulence and spore production. These findings provide new insights into the functional role of TPKs in plant pathogenic fungi, although the true tyrosine kinase activity of *Tpk* gene product remains to be determined.

It has been reported that DNA methyltransferase MoDIM-2 functions in conidial production and appressorium formation by regulation of DNA methylation at transcriptional level in the rice blast fungus. However, MoDIM-2 is dispensable for pathogenicity (Jeon et al., 2015). As shown in this work, an SAM-dependent methyltransferase (*Met*) is required for sporulation and virulence in *C. parasitica*, and its DNA methylation and gene expression level changed significantly in response to hypovirus infection. Therefore, it is speculated that hypovirus infection enhances DNA methylation and suppresses gene expression of the methyltransferase, leading to reduced fungal pathogenicity. Whether and how this SAM-dependent methyltransferase functions in fungal methylation, however, needs to be investigated further.

Taken together, the high-resolution DNA methylation maps for *C. parasitica* and the integrated analysis of epigenomic and transcriptomic data in this study shed light to the relationship between DNA methylation and viral perturbation of fungal epigenetics, laying a new ground for future studies.

## Author contributions

RL carried out the methylome and RNA-seq analysis and wrote the manuscript. SZ constructed the mutant strains and determined the phenotypes. YL and XS participated in confirmation of BS-seq and RNA-seq results. ZW participated in the data analysis. BC designed the study and revised the manuscript. All authors read and approved the final manuscript.

### Conflict of interest statement

The authors declare that the research was conducted in the absence of any commercial or financial relationships that could be construed as a potential conflict of interest.

## Supplementary material

Figure S1 The distribution of mCs in *C. parasitica* chromosomes.

Figure S2 GO enrichment **(A)** and KEGG pathway **(B)** analysis of the differentially expressed genes in *C. parasitica*.

Figure S3 WGBS validation with bisulfite sequencing. In each panel, the left histogram shows the validation results from traditional bisulfite sequencing, while the right shows the WGBS results. Methylation levels of individual cytosine sites located on the genome (indicated on the *x*-axis) are shown on the *y*-axis. Promoter regions for the **(A)** Abh gene, **(B)** Tpk gene, and **(C)** 235052 gene are shown.

Figure S4 Validation of RNA-seq results by qRT-PCR for 6 selected DMR-associated genes. Values were calculated from three independent experiments. Bars show standard error of the mean. Hpt (Hypothetical protein) indicates the gene (75832). The results showed that the EP155 and EP155/CHV1-EP713 expression profiles were consistent with those determined by RNA-seq analysis.

Figure S5 Microscopic examination of strains (the bar represents 20 μm).

Table S1 PCR primers used in this study.

Table S2 Main features of *C. parasitica* genomes for BS-seq.

Table S3 DMR distributions in EP155 and EP155/CHV1-EP713.

Table S4 DMRs in promoter or gene body regions of EP155 and EP155/CHV1-EP713.

Table S5 RNA-seq data for *C. parasitica* EP155 and EP155/CHV1-EP713 strains.

Table S6 Differentially expressed genes in EP155 and EP155/CHV1-EP713.
