# Supplementary figures and images for: Comparative Methylome Analysis Reveals Perturbation of Host Epigenome in Chestnut Blight Fungus by a Hypovirus
Source: Front Microbiol. 2018 May 23;9:1026. doi: 10.3389/fmicb.2018.01026 (PMC5974932; doi:10.3389/fmicb.2018.01026)

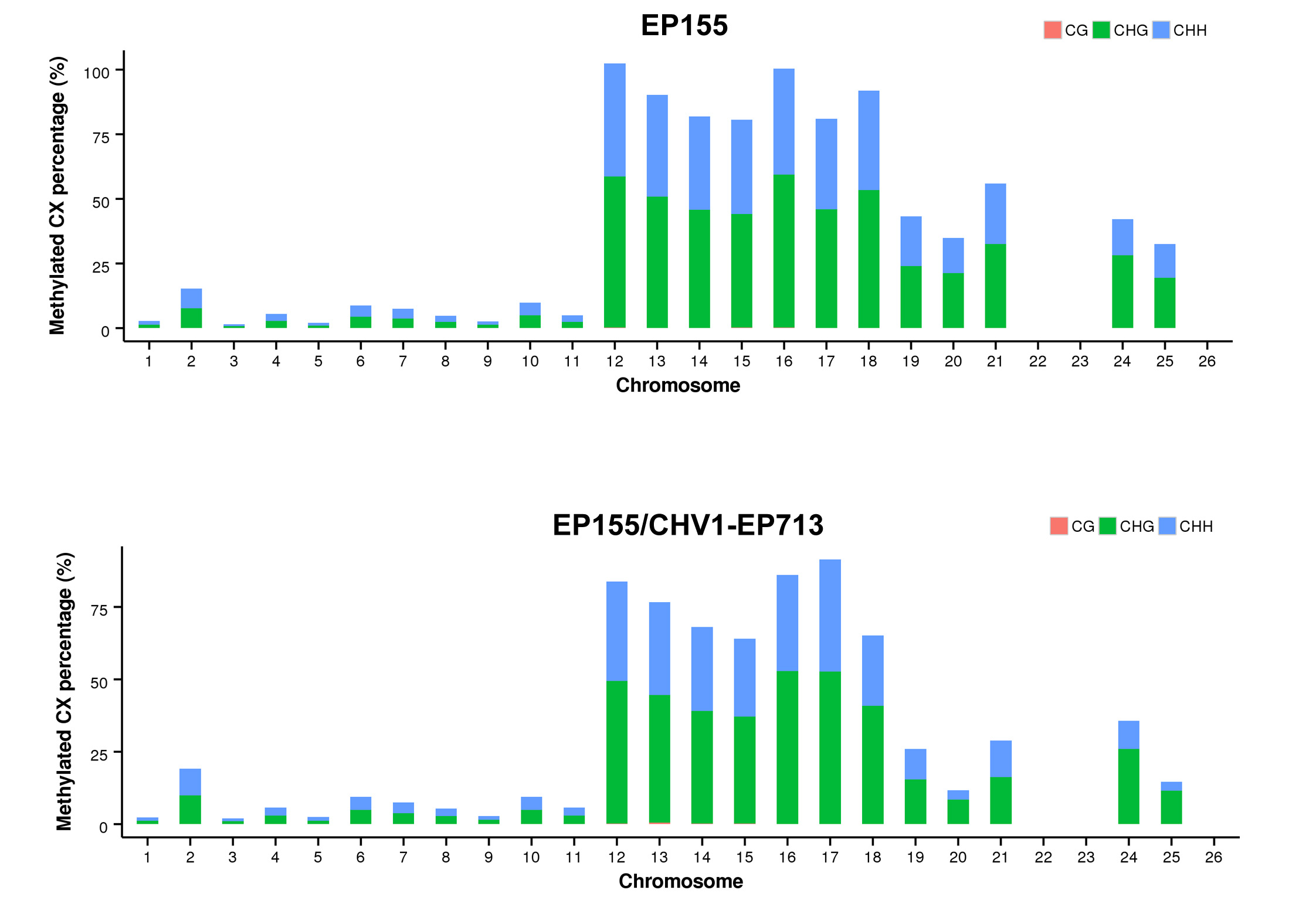

Supplement: Figure S1 — The distribution of mCs in C. parasitica chromosomes. [file Image_1.JPEG]

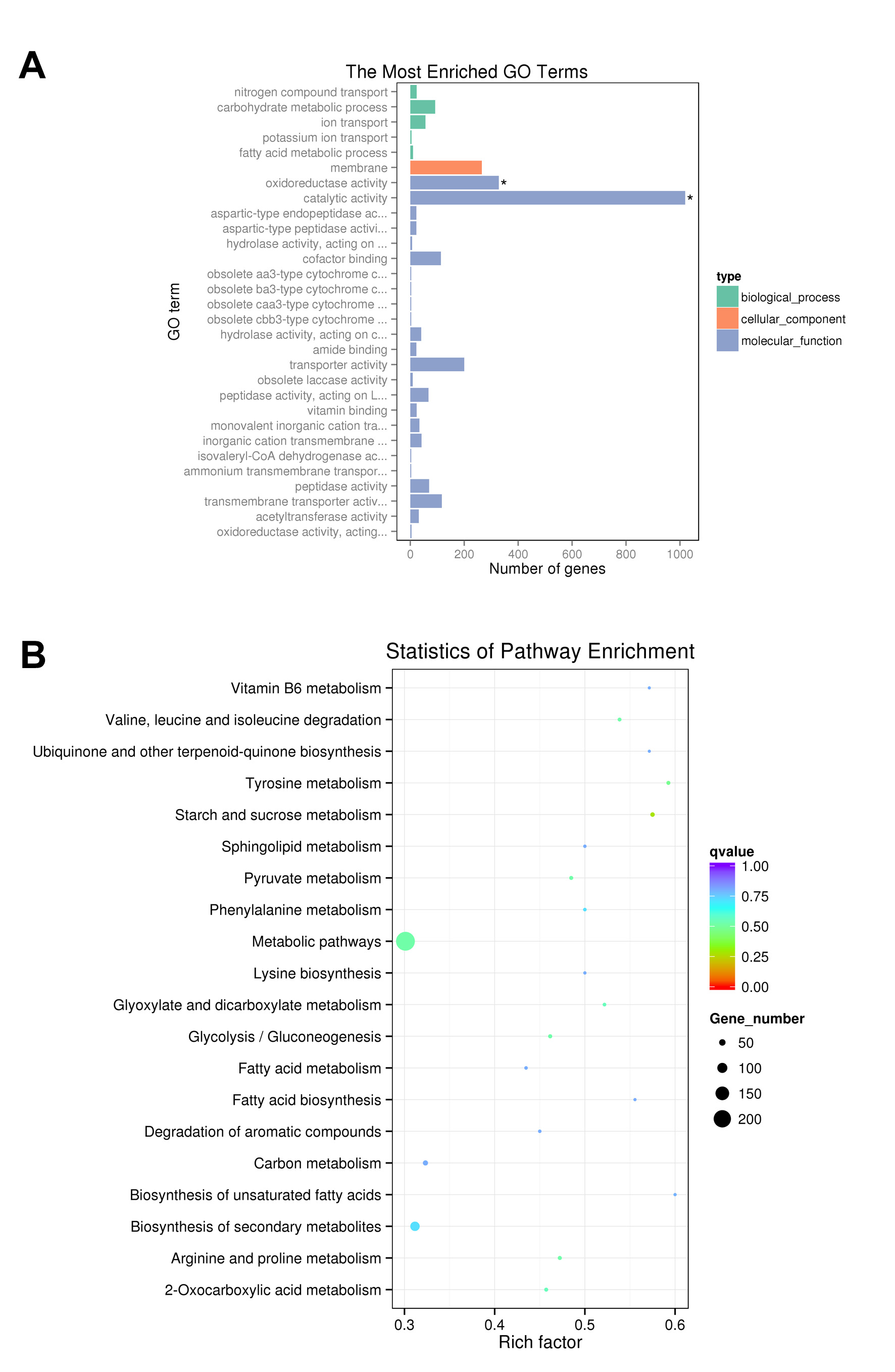

Supplement: Figure S2 — GO enrichment (A) and KEGG pathway (B) analysis of the differentially expressed genes in C. parasitica. [file Image_2.JPEG]

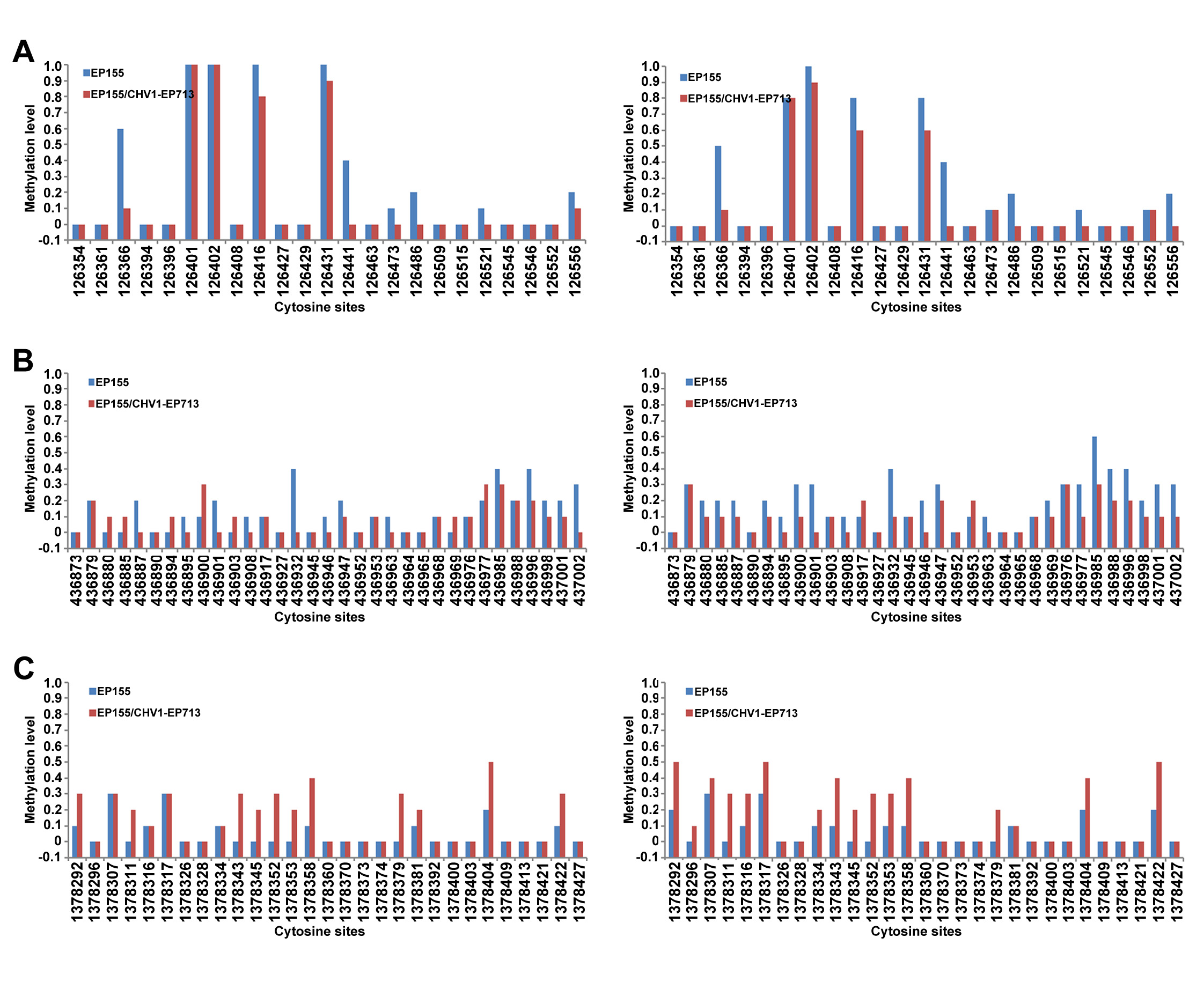

Supplement: Figure S3 — WGBS validation with bisulfite sequencing. In each panel, the left histogram shows the validation results from traditional bisulfite sequencing, while the right shows the WGBS results. Methylation levels of individual cytosine sites located on the genome (indicated on the x-axis) are shown on the y-axis. Promoter regions for the (A) Abh gene, (B) Tpk gene, and (C) 235052 gene are shown. [file Image_3.JPEG]

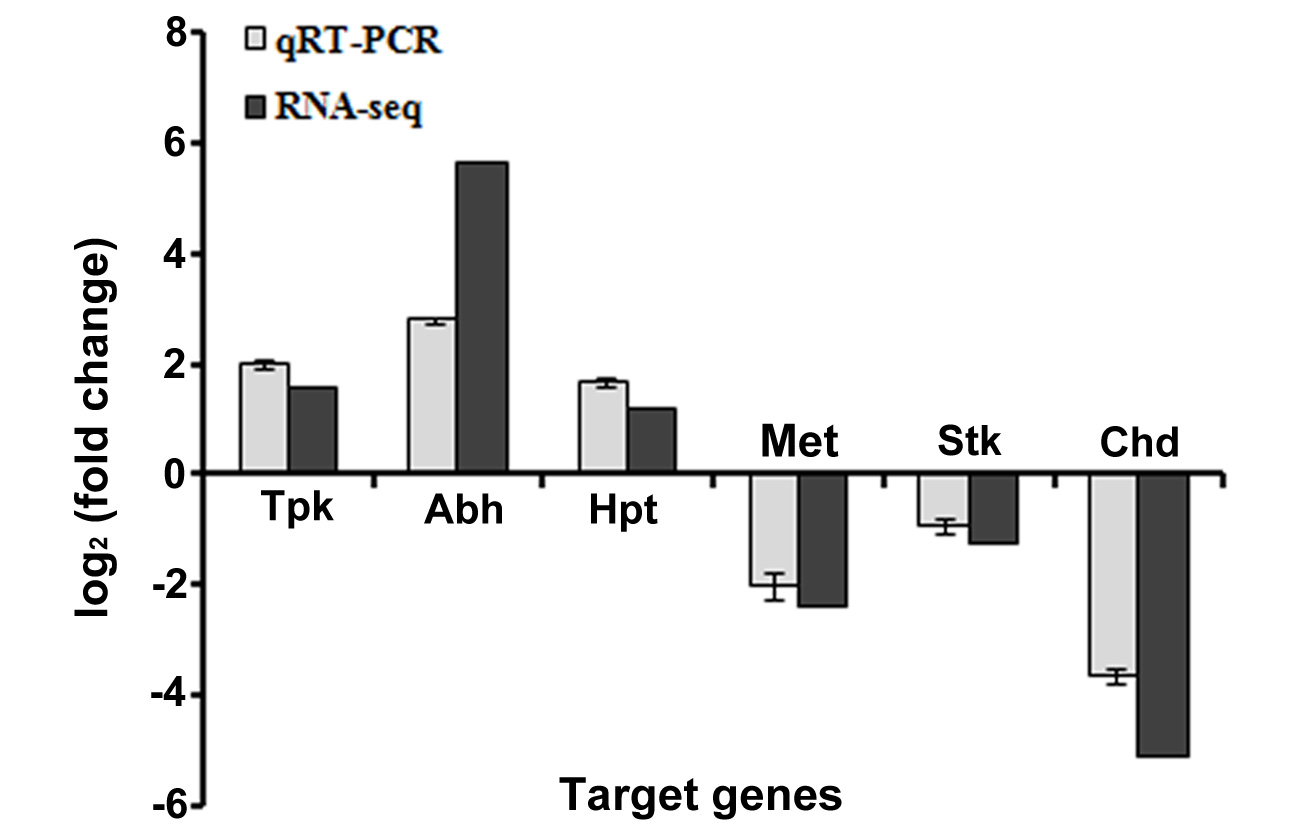

Supplement: Figure S4 — Validation of RNA-seq results by qRT-PCR for 6 selected DMR-associated genes. Values were calculated from three independent experiments. Bars show standard error of the mean. Hpt (Hypothetical protein) indicates the gene (75832). The results showed that the EP155 and EP155/CHV1-EP713 expression profiles were consistent with those determined by RNA-seq analysis. [file Image_4.JPEG]

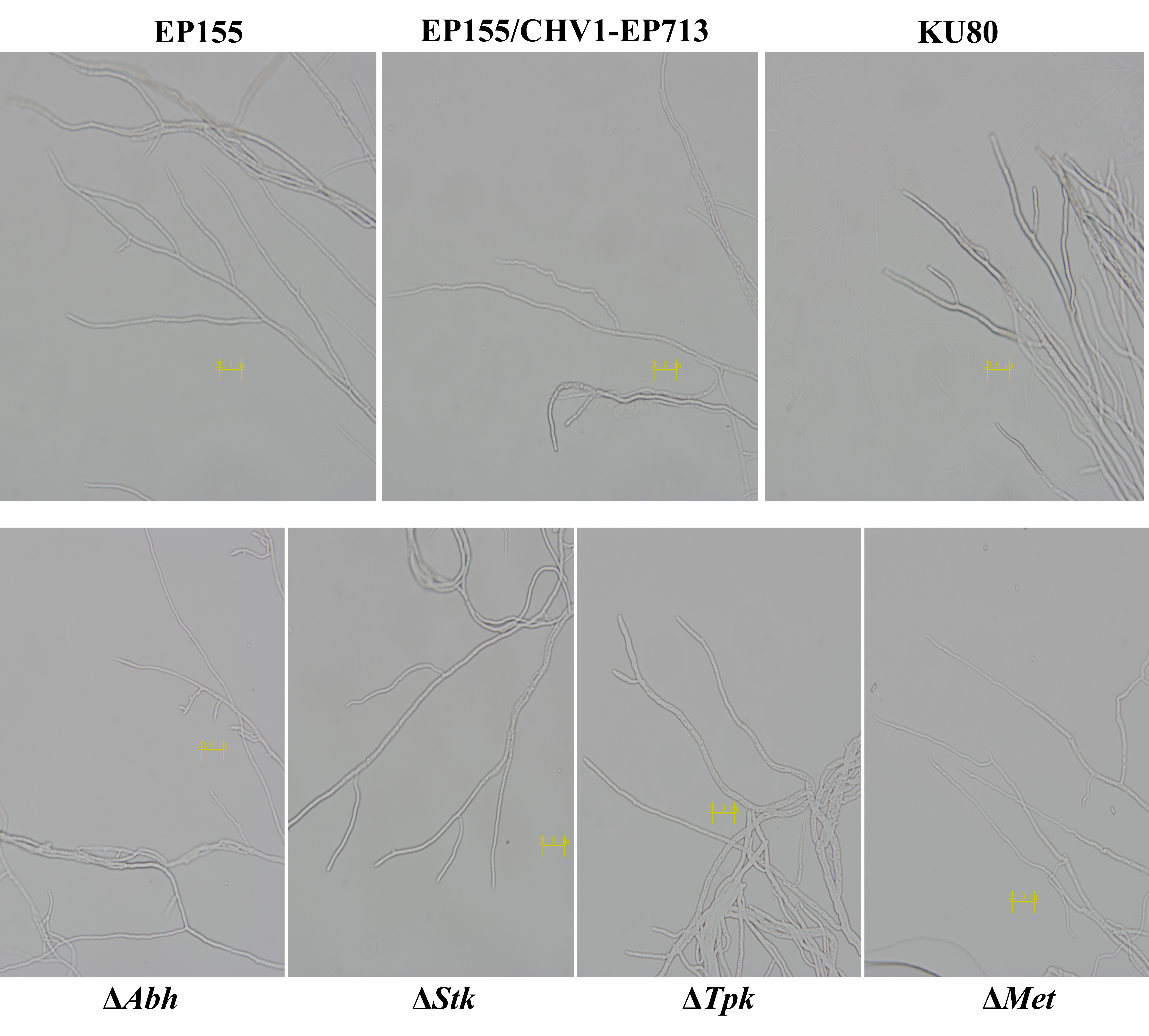

Supplement: Figure S5 — Microscopic examination of strains (the bar represents 20 μm). [file Image_5.JPEG]
